# Recent Advances in the Controlled Release of Growth Factors and Cytokines for Improving Cutaneous Wound Healing

**DOI:** 10.3389/fcell.2020.00638

**Published:** 2020-07-14

**Authors:** Ayan Nurkesh, Alexandr Jaguparov, Shiro Jimi, Arman Saparov

**Affiliations:** ^1^Department of Medicine, School of Medicine, Nazarbayev University, Nur-Sultan, Kazakhstan; ^2^Central Laboratory for Pathology and Morphology, Faculty of Medicine, Fukuoka University, Fukuoka, Japan

**Keywords:** growth factors, cytokines, tissue regeneration, wound healing, biomaterials, controlled release

## Abstract

Bioengineered materials are widely utilized due to their biocompatibility and degradability, as well as their moisturizing and antibacterial properties. One field of their application in medicine is to treat wounds by promoting tissue regeneration and improving wound healing. In addition to creating a physical and chemical barrier against primary infection, the mechanical stability of the porous structure of biomaterials provides an extracellular matrix (ECM)-like niche for cells. Growth factors (GFs) and cytokines, which are secreted by the cells, are essential parts of the complex process of tissue regeneration and wound healing. There are several clinically approved GFs for topical administration and direct injections. However, the limited time of bioactivity at the wound site often requires repeated drug administration that increases cost and may cause adverse side effects. The tissue regeneration promoting factors incorporated into the materials have significantly enhanced wound healing in comparison to bolus drug treatment. Biomaterials protect the cargos from protease degradation and provide sustainable drug delivery for an extended period of time. This prolonged drug bioactivity lowered the dosage, eliminated the need for repeated administration, and decreased the potential of undesirable side effects. In the following mini-review, recent advances in the field of single and combinatorial delivery of GFs and cytokines for treating cutaneous wound healing will be discussed.

## Introduction

Wound healing and tissue regeneration are complex cellular processes that involve an interplay of growth factors (GFs), chemokines, cytokines, and other signaling molecules (Castano et al., 2018; Rodrigues et al., 2018; Brazil et al., 2019). There have been numerous discoveries in the field that enlighten the mechanism of wound healing, and with these scientific advancements, different technological solutions are being suggested. However, a complete understanding of the tissue regeneration mechanisms following wound injury has yet to be deciphered. Worldwide prevalence of skin and subcutaneous diseases are among the most common diseases with just over 600 million people affected in 2015 (Vos et al., 2017). Wound care has a significant economic impact on the world healthcare system and in the United States alone, the cost is $50 billion per year (Gainza et al., 2015b). Medical treatment often cannot completely re-epithelialize the injured site, leaving a scar (Coentro et al., 2018; Monavarian et al., 2019). The scar is a collection of fibrotic cells that in some cases, limit the normal functions of the organ and body (Rippa et al., 2019). Researchers have shown that specific GFs and cytokines play an essential role in cutaneous injury repair and the topical administration of some of them has already proven to significantly enhance wound closure. These GFs include, but are not limited to, epidermal GF (EGF), fibroblast GF (FGF), platelet-derived GF (PDGF), transforming GF-beta (TGF-β), and vascular endothelial GF (VEGF) protein families (Yamakawa and Hayashida, 2019). Growth factors involved in wound healing can be delivered to promote and expedite the process of tissue regeneration. However, the direct application of GFs alone was shown to be ineffective due to the hostile environment at the wound site, subsequent loss of bioactivity of the GFs, and poor skin penetration (Yamakawa and Hayashida, 2019). Additionally, a single injection cannot provide prolonged stimulation of wound healing due to the complexity of the process and repetitive administration can even lead to undesired side effects such as tumorigenesis (Park et al., 2017). Thus, various biomaterials have been used to deliver cytokines and GFs in a controlled fashion. To be effective, biomaterials should be biocompatible, biodegradable, and mimic the composition of the ECM (Mir et al., 2018; Saghazadeh et al., 2018; Suarato et al., 2018; Sultankulov et al., 2019b). They must also be able to bind GFs and cytokines, stabilize them, and provide sustainable release (Elviri et al., 2017; Dellacherie et al., 2019; Wang W. et al., 2019). Apart from that, it has been shown that biomaterials themselves can promote tissue regeneration due to antibacterial, moisturizing, and ECM mimicking properties. This mini-review summarizes recent advances in the development of biomaterials loaded with wound healing stimulating and modulating molecules.

## Hydrogels

One of the most commonly used biomaterials is hydrogel, a polymer network capable of absorbing water and serving as a scaffold for drug delivery, which has been widely used in wound healing (Li and Mooney, 2016; Dimatteo et al., 2018; Daly et al., 2019). Since the end of the 20th century, FGF family proteins have been attractive in drug development and one have been clinically approved for wound treatment (Hui et al., 2018a). Basic FGF (bFGF) incorporated into a bioinspired hydrogel demonstrated a sustained release of GF over time, increased fibroblast proliferation *in vitro*, as well as enhanced wound closure in the scratch assay, and promoted wound healing in the full-thickness skin incision murine wound model (Zhang et al., 2018). In addition, bFGF released from hydrogel promoted higher fibroblast and extracellular protein density. It is important to note that hydrogels were used as a drug delivery system to stabilize the embedded GFs from rapid protease degradation at ECM, and served as a supportive material to enhance wound healing. In the case of acidic FGF (aFGF)-Carbomer 940 hydrogel, higher stability of aFGF in comparison to bolus aFGF, which was used in a non-diabetic murine wound model, resulted in accelerated cell proliferation, neo-vascularization, and wound healing in type 2 diabetic rats (Hui et al., 2018b). In some studies, the hydrogel was incorporated with other drug delivery systems such as microspheres and liposomes (Xu et al., 2017, 2018; Shamloo et al., 2018). This double layer system promoted the sustained release of the incorporated bFGF for a longer period of time, resulting in enhanced wound closure, organized collagen deposition, vascularization, granulation, myofibroblast formation, and reduced inflammation. The proposed mechanism for increased neovascularization was through bFGF induced VEGF expression and better healing was also due to deeper permeation of bFGF in the wounded area (Xu et al., 2017, 2018). Another research group demonstrated that hyaluronan/collagen hydrogel containing heparin-binding EGF-like GF enhances wound healing in an organ culture model of the porcine skin (Thönes et al., 2019). Heparin also enabled sustained release of stromal cell-derived factor-1 (SDF-1) that accelerated wound healing and increased vascularization in rats (Yao et al., 2020). In order to maximize protection from degradation, Devalliere et al. (2017) constructed genetically fused proteins from elastin-like peptide (ELP) with keratinocyte GF (KGF) or ARA290 (Erythropoietin derivative), an apoptosis-protective molecule, loaded them into fibrin hydrogel and tested their combinatorial effect on skin wounds of diabetic mice (Devalliere et al., 2017). The results demonstrated improved wound healing by facilitating blood vessel formation, epithelialization, and granulation tissue formation. It was suggested that ELP promoted dermis regeneration and limited accessibility of the GFs for protease degradation and collectively ELP-KGF stimulated keratinocytes migration and proliferation, whereas ELP-ARA290 assisted in inhibiting tissue apoptosis.

Another approach is to use hydrogel in its liquid phase for better coverage of the injured site. One group used a heparin-poloxamer consisting hydrogel, which is liquid at 4°C and solid at body temperature. The time of the topical administration was just enough to cover the wounded area tightly prior to formation of the gel substance. Wu et al. (2016b) also used a thermo-sensitive hydrogel to show that the binding interaction between drug and scaffold is crucial by comparing the effectiveness of hydrogel loaded with aFGF or bFGF. In addition to overall improvement in wound healing, the authors demonstrated that the structural differences between GFs of the same family affected the efficiency of tissue regeneration. Even though both GFs were linked to the material via their heparin-binding domain, aFGF demonstrated to be more effective than bFGF. An interesting approach took advantage of the increased temperature at the inflammation site for controlled delivery of VEGF using a thermosensitive microneedle hydrogel. This study demonstrated enhanced wound healing, granulation, collagen deposition, angiogenesis, and reduced inflammation (Chi et al., 2020). Similarly, other groups invented a self-healing type of hydrogel that behaved as a liquid during syringe injection and solidified upon wound coverage (Chen G. et al., 2017; Chen et al., 2019). The self-healing properties of the hydrogel particles were attributed to the imine and acylhydrazone bonds (Chen et al., 2019). The use of this type of hydrogel, with loaded bFGF, enhanced the formation of granulation tissue, increased angiogenesis, and inhibited inflammation and activity of the pro-inflammatory factors, TNFα and IL-6 (Chen G. et al., 2017). Other studies evaluated the effect of an injectible hydrogel in complex with silver and bFGF on infected wounds. The results demonstrated the advantages of a composite by observing improved wound closure, collagen deposition, vascularization, re-epithelialization, granulation, and keratinocytes migration. In addition, reduced inflammation was associated with the anti-inflammatory M2 but not pro-inflammatory M1 macrophages (Xuan et al., 2020).

The natural wound healing process never comes with a single molecule orchestrating the activity of the downstream effectors, but rather a complex interplay of all components at specific stages (Rousselle et al., 2018). A further improvement toward the simulation of the programmed molecular “game” of the living organism is the delivery of two or more active molecules. Several groups have used combinations of different GFs or GFs with active molecules (Yang et al., 2017; Park et al., 2018; Yoo et al., 2018). Yoo et al. (2018) studied the combination of EGF and bFGF incorporated into hydrogel. Increased rate of wound closure, decreased number of pro-inflammatory cells, enhanced re-epithelialization, and granulation tissue formation were associated with the dual effect of EGF and bFGF. It was suggested that EGF promoted fibroblast migration, while bFGF was responsible for enhanced collagen deposition, granulation, and re-epithelialization by secreting TGF-α and IL-1 (Yoo et al., 2018). Another group demonstrated enhanced wound healing by dual delivery of Substance-P and TGF-β1 loaded microparticles in the injectable hydrogel to the irradiated skin of mice. Specifically, nude mouse panniculus adiposus and carnosus layers were thicker and repaired faster in the dual delivery group compared to the control group. It’s been proposed that the drug’s effect was mediated by the migration of mesenchymal stem cells (Park et al., 2018). Thus, thermo-sensitive hydrogels alone, or in combination with microvesicles, have demonstrated the ability to enhance wound healing by protecting proteins from protease degradation and promoting gradual release of the drugs over an extended period of time. Moreover, studies showed that dual drug delivery was more effective than a single drug incorporated hydrogel. Table 1 summarizes the effects of GFs and cytokines on wound healing.

**TABLE 1:** Effects of growth factors and cytokines on wound healing.

**Material**	**Growth factor/cytokine**	**Effect**	**References**
Hydrogel	bFGF	Increased fibroblast proliferation *in vitro*, enhanced wound closure in the scratch assay, and promoted wound healing *in vivo*	Zhang et al., 2018
	bFGF	Enhanced wound closure, organized collagen deposition, vascularization, granulation, myofibroblast formation, and reduced inflammation	Xu et al., 2017, 2018
	bFGF	Enhanced the formation of granulation tissue, increased angiogenesis, and inhibited inflammation and activity of the pro-inflammatory factors, TNFα and IL-6	Chen G. et al., 2017
	bFGF	Enhanced wound closure, collagen deposition, vascularization, re-epithelialization, granulation, keratinocytes migration, and reduced inflammation	Xuan et al., 2020
	aFGF	Accelerated cell proliferation, neo-vascularization, and wound healing	Hui et al., 2018b
	aFGF or bFGF	Improved wound healing	Wu et al., 2016b
	EGF and bFGF	Increased rate of wound closure, decreased number of pro-inflammatory cells, enhanced re-epithelialization, and granulation tissue formation	Yoo et al., 2018
	SDF-1	Accelerated wound healing and increased vascularization	Yao et al., 2020
	VEGF	Enhanced wound healing, granulation, collagen deposition, angiogenesis, and reduced inflammation	Chi et al., 2020
	EGF-like growth factor	Enhanced wound healing in an organ culture model of the porcine skin	Thönes et al., 2019
	ELP-KGF and ELP-ARA290	Improved wound healing by facilitating blood vessel formation, epithelialization, and granulation tissue formation	Devalliere et al., 2017
	Substance-P and TGF-β1	Enhanced wound healing, thicker panniculus adiposus and carnosus layers	Park et al., 2018
Nanoparticle	KGF	Enhanced wound closure and cell migration	Muhamed et al., 2019
	KGF	Enhanced wound healing and re-epithelialization, accelerated keratinocyte migration and proliferation	Pan et al., 2018
	KGF	Enhanced wound healing, bioactivity, and increased levels of collagen I, α-SMA, and TGF-β1	Li et al., 2019
	EGF	Accelerated migration of inflammatory cells during the inflammation phase, enhanced wound healing	Kang et al., 2017
	EGF	Enhanced wound closure, collagen deposition, angiogenesis, and reduced inflammation and number of myofibroblasts	Gainza et al., 2015a
	VEGF164	Enhanced wound closure accompanied by increased re-epithelialization and granulation but not wound contraction	Chereddy et al., 2015
Nanofiber	G-CSF	Improved wound closure, scar reduction, fibroblast maturation, as well as increased collagen density and decreased amount of neutrophils	Tanha et al., 2017
Biofilm	bFGF	Enhanced healing, reduced pro-inflammatory cell accumulation, promoted granulation layer formation	Liu et al., 2017
	EGF	Accelerated wound healing via stimulating keratinocyte differentiation and reducing inflammation	Kim et al., 2016
	VEGF and PDGF-BB	Enhanced angiogenesis, granulation, and keratinocyte proliferation	Almquist et al., 2015
Coacervate	bFGF	Enhanced wound closure, angiogenesis, collagen deposition, granulation, cell proliferation in the wound area, re-epithelialization, and hair follicle formation	Wu et al., 2016a
	IL-10 and TGF-β3	Accelerated wound closure, enhanced re-epithelialization, angiogenesis, collagen I distribution, and reduced hypertrophic scar formation	Park et al., 2019
Fibrin matrix	VEGF-A165 and PDGF-BB	Enhanced vascularization, suppressed migration of the neutrophils to the wounded area, and attracted Ly6C^+^CD11b^+^ monocytes	Ishihara et al., 2018
Collagen matrix	EGF or bFGF	Accelerated wound healing, re-epithelialization, neovascularization, and collagen deposition	Choi et al., 2018
Lyotropic liquid crystal	EGF	Reduced inflammation, increased wound closure and re-epithelialization	Zhou et al., 2019
	VEGF	Enhanced vascularization	Wang B. et al., 2019
Cryogel	IL-10, TGF-β1, VEGF and bFGF	Improved the regenerative process on a murine internal splint wound model, including neovascularization, wound closure, granulation, and re-epithelialization	Jimi et al. (2020)

## Nanoparticles

Nanoparticles possess a wide range of applications, including usage in drug administration (Patra et al., 2018). A number of papers have been published that use drug loaded nanoparticles in treating wounds. For example, fibrin nanoparticles loaded with KGF enhanced wound closure and cell migration *in vivo* by binding KGF to its receptor located on the keratinocyte (Muhamed et al., 2019). Other groups showed the advantage of nanoparticles based delivery over GF/cytokine treatment alone. Application of the hyaluronic acid microparticles loaded with EGF to the wound area enabled a similar effect of bolus EGF administration with significantly lower concentration. Moreover, accelerated migration of inflammatory cells during the inflammation phase enhanced wound healing (Kang et al., 2017). KGF linked gold nanoparticles (GNP) exhibited enhanced wound healing and re-epithelialization properties in comparison to KGF administration alone. There was an acceleration in keratinocyte migration and proliferation, but no significant promotion in granulation layer formation, which contributes to extensive fibrosis (Pan et al., 2018). Furthermore, the same research group investigated the effect of KGF loaded GNPs on a diabetic mouse wound model and confirmed previous findings. In addition, they confirmed that KGF-GNP specifically binds to KGF receptor and even better than KGF alone, possesses a higher level of resistance to harsh environments, and increases levels of collagen I, α-SMA, and TGF-β1, which are associated with the wound healing process (Li et al., 2019).

Non-diabetic and diabetic murine skin wounds treated with VEGF164 loaded poly(lactic-co-glycolic acid) nanoparticles showed enhanced wound closure accompanied by increased re-epithelialization and granulation but not wound contraction. The suggested mechanism was via VEGFR2 activation which is known to be a mediator of VEGF for angiogenesis through further stimulation of p38/MARK pathway protein, kinase B for apoptosis inhibition and cell proliferation (Chereddy et al., 2015; Aday et al., 2017). Gainza et al. (2015a) used a porcine wound healing model and demonstrated that wound healing was enhanced by the application of EGF loaded lipid nanoparticles in terms of enhanced wound closure, collagen deposition, angiogenesis, and reduced inflammation and number of myofibroblasts. However, the study shows that bolus EGF treatment also resulted in enhanced wound healing and the authors explain the advantages of nanostructure linked GF by reduced concentration (Gainza et al., 2015a).

Another example of a drug delivery system with promising therapeutic applications are nanofibers. Nanofibrous coating is a porous dressing that better simulates an ECM structure (Gainza et al., 2015b; Chen S. et al., 2017). One group used granulocyte colony-stimulating factor (G-CSF) incorporated into chitosan nanoparticles and further mixed with poly(ε-caprolactone) (PCL) nanofibers. Moreover, to increase cell attachment, the scaffold was coated with collagen I. *In vivo* experiments demonstrated improved wound closure, scar reduction, fibroblasts maturation, as well as increased collagen density and a decreased amount of neutrophils (Tanha et al., 2017).

## Other Drug Delivery Systems

The last section is dedicated to other developed or advanced drug delivery systems for wound healing. Both chitosan and collagen mentioned above were also used in biofilm composition, which is another type of drug delivery system. For the mechanical stability of the structure, authors used grapheme oxide (GO) rather than nanofibers by considering its physicochemical properties and an increasing interest for wide applications in biomedicine. A modified collagen-chitosan biofilm, which was loaded with bFGF, showed enhanced healing in full-thickness wounds in Sprague-Dawley rats by reducing pro-inflammatory cell accumulation and promoting granulation layer formation (Liu et al., 2017). Deeper penetration was also responsible for wound acceleration in hyaluronate (HA) conjugated EGF film administered on a rat wound model. Mechanistically, HA elevated β-defensin 2 activity stimulated keratinocytes differentiation, whereas the increased levels of TGF-β contributed to cellular proliferation, and diminished levels of TNF-α and IL-1 were associated with reduced inflammation (Kim et al., 2016). The concentration dependent effect was observed using VEGF with PDGF-BB embedded multilayer film that enhanced angiogenesis, granulation (combinatorial effect), and keratinocyte proliferation (Almquist et al., 2015).

One example is self-assembled hydrophobic vehicle particles composed of different materials collectively named as coacervates. Heparin composed coacervate, which was loaded with bFGF, enhanced wound closure, angiogenesis, collagen deposition, granulation, cell proliferation in the wound area, re-epithelialization, and hair follicle formation. These results were associated with increased levels of TGF-β1, CD31, and α-SMA. Additionally, VEGF activity was also promoted by bFGF in the first 7 days post-skin injury consistent with other reports (Wu et al., 2016a). Another group used heparin for dual delivery of GFs utilizing nanofibers coated with coacervate made of poly (ethylene argininyl aspartate diglyceride) and heparin. IL-10 and TGF-β3 bind to heparin via its negatively charged groups (Park et al., 2019). The combinatorial delivery of IL-10 with TGF-β3 on a rat skin wound model showed accelerated wound closure, enhanced re-epithelialization, angiogenesis, collagen I distribution, and reduced hypertrophic scar formation. TGF-β can mediate its effect through activation of Smad proteins (Landén et al., 2016).

Ishihara and colleagues demonstrated that GFs can also specifically bind to laminin, an ECM component, and then promote wound healing. VEGF-A165 and PDGF-BB selectively bind to the heparin-binding domain of the laminin embedded into the fibrin matrix. The complex further enhanced vascularization, suppressed migration of the neutrophils to the wounded area, and attracted Ly6C + CD11b + monocytes (Ishihara et al., 2018). Another distinctive approach was achieved by Choi and colleagues via the addition of disulfide bonds to EGF and bFGF. Moreover, the authors separately incorporated these GFs into a collagen based matrix and demonstrated accelerated wound healing, re-epithelialization, neovascularization, and collagen deposition in type I and type II diabetic mice wound models (Choi et al., 2018).

Lyotropic liquid crystals (LLC) possess gelation properties as injectable hydrogels and form a solid gel-like substance upon exposure to liquid at the wounded area. Several studies observed reduced inflammation, increased wound closure and re-epithelialization with EGF loaded LLC, and well developed blood vessels with VEGF loaded LLC using the skin wound excisional mice models in both cases (Wang B. et al., 2019; Zhou et al., 2019).

Based on our previously published studies on successful simultaneous incorporation of perivascular stem cell secreting three GFs, VEGF, MCP-1, and IL-6, into heparin coacervate (Mansurov et al., 2017), and the use of a composite cryogel for GF delivery (Sultankulov et al., 2019a), our group tested the sequential topical application of cryogel that was loaded with four GFs/cytokines. The sequential targeted delivery of cryogel released IL-10 and TGF-β1 that was followed by the delivery of VEGF and bFGF significantly improved the regenerative process on a murine internal splint wound model, including neovascularization, wound closure, granulation, and re-epithelialization (Jimi et al., 2020).

## Conclusion

A number of studies demonstrated that clinically approved GFs affect the proliferation stage of wound healing by regulating tissue regenerating and immunomodulatory signaling pathways. However, the low stability and hence short time of the protein’s bioactivity at the site of wound injury limits the effectiveness of bolus administration. Development of biocompatible materials has significantly enhanced tissue regeneration and wound healing. Biomaterials composed of a hydrogel, nanoparticles, nanofibers, cryogel, and a combination of other molecules served as a drug delivery system by protecting GFs/cytokines from protease degradation and promoting sustained release over an extended period of time. The major challenges for drug loaded biomaterials are mechanical strength and porosity for simulation of the ECM, ability to gradually release the linked cargos and biodegradability. In addition, some research groups demonstrated that the combinatorial effect of the simultaneous delivery of two or more GFs/cytokines promotes tissue regeneration even further, thus revealing the advantage of a combinatorial effect in contrast to single drug administration. Overall, the rapidly growing field of drug delivery systems for wound healing has an important value for their therapeutic application in medicine.

## Author Contributions

AS contributed to the conception of the study and edited the manuscript. AN, AJ, and SJ wrote the sections of the manuscript. All authors read and approved the submitted version.

## Conflict of Interest

The authors declare that the research was conducted in the absence of any commercial or financial relationships that could be construed as a potential conflict of interest.
